# Microsurgical treatment for parasagittal meningioma in the central gyrus region

**DOI:** 10.3892/ol.2013.1429

**Published:** 2013-06-26

**Authors:** NA BI, RUI-XUE XU, RONG-YAO LIU, CHUN-MING WU, JIAN WANG, WEI-DONG CHEN, JUN LIU, YOU-SONG XU, ZHEN-QING WEI, TAO LI, JIAN ZHANG, JING-YANG BAI, BIN DONG, SHU-JUN FAN, YING-HUI XU

**Affiliations:** 1 Department of Nursing, Chinese PLA General Hospital, Beijing 100853, P.R. China; 2 Department of Neurosurgery, The 309th Hospital of PLA, Beijing 100091, P.R. China; 3 Department of Neurosurgery, The First Affiliated Hospital of Dalian Medical University, Dalian, Liaoning 116044, P.R. China; 4 Department of Pathology, Dalian Medical University, Dalian, Liaoning 116044, P.R. China

**Keywords:** parasagittal meningioma, central gyrus region, microsurgery, Rolandic vein

## Abstract

The aim of the present study was to determine the efficacy of microsurgery treatment for parasagittal meningioma in the central gyrus region. A microsurgical technique was used to treat 26 patients with large parasagittal meningioma in the central gyrus region. The Rolandic and draining veins and the peritumoral normal brain tissue were retained, and the associated sagittal sinus was appropriately protected. A Simpson grade I, II or III resection was performed in 8 (30.8%), 12 (46.2%) and 6 (23.1%) patients, respectively, with no post-operative mortalities. Following treatment, 9 patients exhibited hemiparalysis. No tumor recurrence was found in 21 patients during the follow-up examination. The treatment protocol described in the current study included sufficient pre-operative imaging evaluations, a skilled microsurgical technique, improved protection of the Rolandic vein and treatment of the sagittal sinus, and was found to significantly increase the total tumor removal rate and decrease post-operative recurrence.

## Introduction

Meningiomas are the most common intracranial tumors and include convex, parasagittal, sphenoid ridge and cerebellopontine angle meningiomas. Parasagittal meningiomas account for the 17–20% morbidity rate of intracranial meningiomas and the 33% morbidity rate of parasagittal and falx meningiomas (1). Surgical resection is the first choice of treatment for meningiomas. As the paracentral lobule, central lobe, draining vein, venous sinus and other important tissues are involved in the microsurgical treatment of parasagittal meningiomas, serious complications may occur following tumor resection. The neural function of patients may be affected, resulting in a reduction of their quality of life. Therefore, the improvement of surgical techniques is vital for enhancing the quality of life of meningioma patients (2,3). In recent years, microscopic techniques in neurosurgery have progressed and the efficacy of these techniques has been improved by the development of suitable surgical strategies. In the present study, 26 patients with large parasagittal meningioma in the central gyrus region were treated with microsurgery, and the efficacy of this technique is discussed.

## Materials and methods

### Patients

A total of 26 patients (17 males and 9 females) with large parasagittal meningioma in the central gyrus region who were admitted to the First Affiliated Hospital of Dalian Medical University (Dalian, Liaoning, China) were studied between 2004 and 2010. This study was conducted in accordance with the Declaration of Helsinki and was approved by the Ethics Committee of the First Affiliated Hospital of Dalian Medical University. Written informed consent was obtained from all participants. The patient age ranged between 23 and 68 years, with an average age of 46.2 years. Their clinical courses ranged between 1.5 months and 10 years, with an average of 10.2 months. Of the 26 patients, 3 had been diagnosed with a post-operative recurrence of meningioma.

### Clinical symptoms

Of the 26 patients, all reported various degrees of dizziness and headache, 10 presented with epilepsy, 15 with lateral limb numbness, 16 with hemiparesis, 2 with light coma and 8 with a positive pathological reflex.

### Imaging examination

An X-ray examination was conducted in 10 patients and there were 8 cases with local cranial hyperostosis. In the 23 patients examined by computed tomography (CT), the central gyrus sinus presented with a high-density shadow that was round in shape, with clear boundaries, marked enhancement and surrounding edema. Certain patients exhibited cranial hyperostosis. Magnetic resonance imaging (MRI) was performed in all 26 patients. The equal T1 or slightly high T1, high T2 or slightly high T2 signals were found with clear boundaries, marked enhancement and surrounding edema in the lesion site. The maximum diameter of the tumor ranged between 4.0 and 9.6 cm, with an average diameter of 5.6 cm. Digital subtraction angiography (DSA) was used to examine 12 patients that presented with large tumors with a rich blood supply. The blood was being supplied to the parasagittal meningiomas by the external and internal carotid arteries. Arterial embolization was performed in 3 patients with scalp vascular dilatation. Of the 26 patients, there were 18 cases with lateral tumors, 8 cases with bilateral tumors crossing the sagittal sinus, 16 cases with tumors crossing the Rolandic vein and 10 cases with tumors oppressing the Rolandic vein.

### Microsurgery method

The patients lay in the supine position with their head fixed in a Mayfield head frame. The microsurgery was conducted as follows: i) A midline horseshoe-shaped incision was performed for adequate exposure of the frontal and posterior boundary of tumor. ii) The dura mater was cut open along the tumor edge, avoiding damage to the draining vein. Following gradual reduction of the tumor volume by intratumoral block excision, the tumor capsule was separated. The tumor tissue outside the sagittal sinus was removed, then the tumor tissue inside the sagittal sinus and the invaded sagittal sinus wall were treated. iii) Tumors crossing the Rolandic vein were removed by block excision to protect the Rolandic vein. Following a reduction of the tumor size, the Rolandic vein was stripped. A small amount of tumor tissue may have remained as it was extremely difficult to strip. The peritumoral thick vein for compensatory backflow was protected. iv) In patients with sagittal sinus blockage, the blocked region was removed following ligation. In cases where the tumor was tightly adhered to the sagittal sinus, bipolar electrocautery was conducted on the tumor tissue and sagittal sinus wall. During the surgical procedure, the electric coagulation power was controlled and the temperature was lowered with continued flushing water. v) The eroded skull and meninges were removed. In cases where the skull was minimally eroded, the inner plate and diploe were resected without repair.

## Results

### General information

According to the Simpson grading scale of tumor resection (4), Simpson grade I, II or III resection was performed in 8 (30.8%), 12 (46.2%) and 6 (23%) of 26 patients, respectively, with no perioperative mortalities. In the 16 cases of pre-operative hemiplegia, 9 patients exhibited temporary post-operative aggravated hemiplegia or lower limb paralysis.

### Microsurgery effect

**Figure 1:**
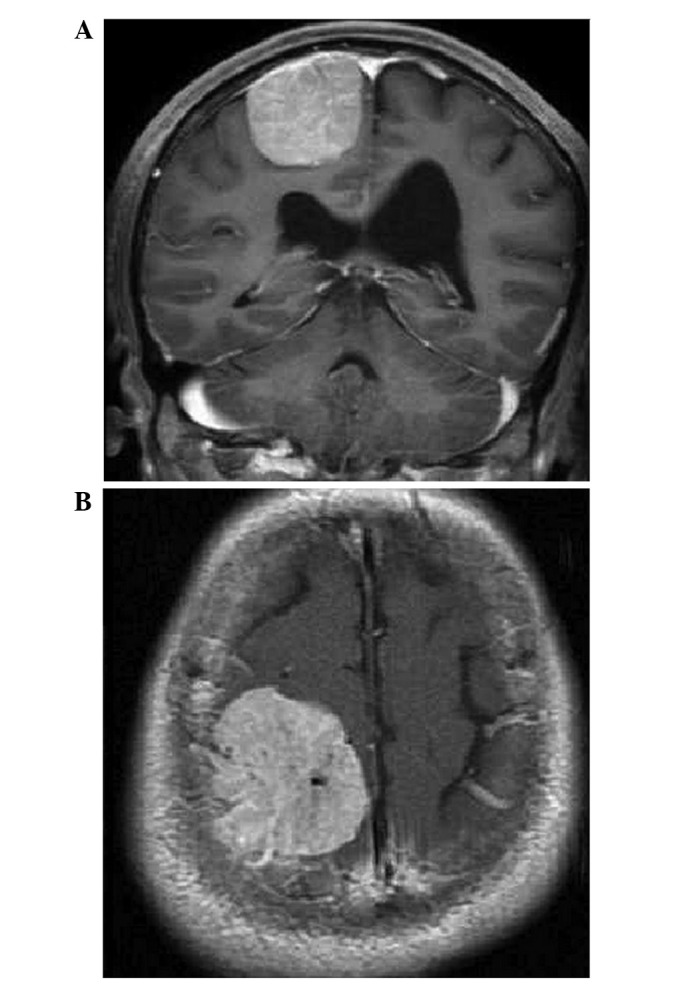
Pre-operative magnetic resonance imaging (MRI) of parasagittal meningioma in the central gyrus region. (A) coronal position; (B) horizontal position.

**Figure 2:**
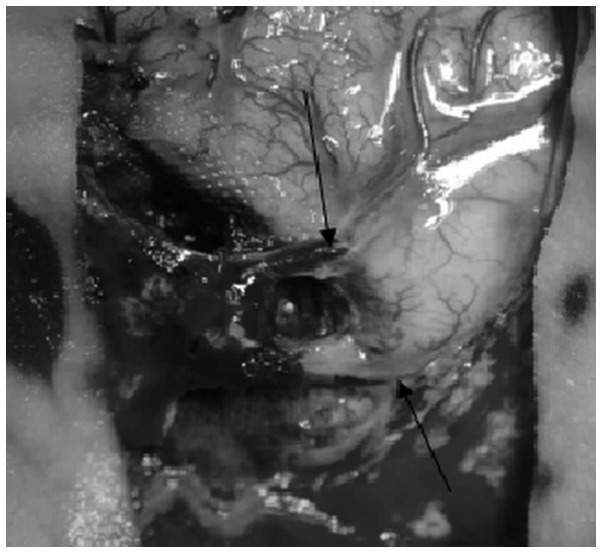
Image of the central gyrus following the complete resection of a parasagittal meningioma. Arrows indicate the preserved Rolandic vein and draining vein.

**Figure 3:**
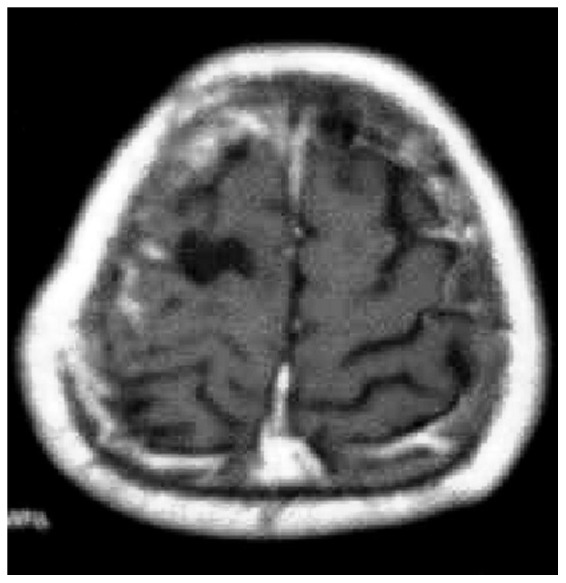
Post-operative magnetic resonance imaging (MRI) of a parasagittal meningioma in the central gyrus region.

Prior to microsurgery, MRI showed parasagittal meningioma in the central gyrus region (Fig. 1). Following the total resection of the parasagittal meningioma, the central sulcus veins and superior cerebral veins flowed under the dura mater for a distance of 2 cm, then back flowed to the superior sagittal sinus (Figs. 2 and 3). This indicated that, the central sulcus vein and superior cerebral veins were well retained. Following active treatment, 6 patients recovered to normal and 3 patients did not recover (1 case of grade III and 2 cases of grade II) upon discharge from hospital. In these 3 patients, 1 case (grade III) had recovered at the four-month follow-up and the other 2 cases had not fully recovered, but had taken a favorable turn (grade III). The pathology reports revealed that the number of patients with endothelial cell-, fiber cell-, psammoma body-, vascular- and mixed-type tumors was 1, 7, 4, 2 and 1, respectively. No post-operative recurrence was found in 21 patients during the follow-up period, which ranged between 8 months and 5 years (average, 20.3 months).

## Discussion

At present, the treatment procedure for parasagittal meningioma requires the maximization of tumor tissue removal, as well as improved protection of the central gyrus brain tissue, Rolandic vein and draining vein, and the effective treatment of the sagittal sinus (2).

Following the development of modern imaging technologies and the gradual popularization of CT, MRI and DSA, parasagittal meningiomas in the central gyrus region may now be clearly determined and qualitatively diagnosed. However, due to the position of the tumor, the associated sagittal sinus and draining vein may be damaged during resection of the tumor, leading to limb paralysis, severe brain edema and even mortality. The surgical treatment of parasagittal meningiomas remains difficult, and methods to maximize the removal of the tumor tissue and minimize the surgical damage must be developed. Pre-operative imaging examinations to determine the location, shape, size and blood supply of the parasagittal meningioma and the relationship between the tumor and peritumoral tissue and vessels is important during medical examinations (3,5–7). Imaging also provides information to aid clinicians in reducing the surgical risk and the tumor blood supply via embolization (8). CT and MRI examinations are used to visualize the tumor location, shape and size. MR angiography (MRA), DSA and CT angiography (CTA) are utilized to determine the position of the Rolandic vein and tumor blood supply and the status of the sagittal sinus and collateral circulation (7,10). These methods are useful for the reduction of intraoperative bleeding and injury, the protection of the draining vein and the effective treatment of the associated sagittal sinus.

In the present study, a DSA examination was performed on 12 patients with large tumors (diameter, >6 cm), which enabled the generation of a comprehensive understanding of the tumor blood supply and the states of the sagittal sinus and peritumoral veins. Pre-operative external carotid artery embolization was performed on 3 patients with a rich blood supply from an enlarged external carotid artery. This procedure may be used to reduce the tumor blood supply and improve the surgical success rates.

The external and internal carotid arteries provide the main blood supply to parasagittal meningiomas, and the diploe and meningeal veins are involved in drainage (2,11,14). When intracranial pressure is high, the draining vein and sagittal sinus are compressed, and intracranial vein blood flows out through the diploe vein, increasing scalp, skull and endocranial hemorrhaging during craniotomy. In cases where the tumor tissue adheres to the skull inner plate, opening the bone flap is extremely difficult and blood loss is likely to be ≥1,000 ml. Therefore, the control of bleeding during craniotomy is extremely important for the success of surgical procedures and for improving the prognosis.

In clinical practice, the measures for controlling bleeding are as follows (2,8,9): i) Patients lie in the head-up position and hyperventilation is conducted under anesthesia. ii) For tumors with a rich blood supply from the external carotid artery, the end of the superficial temporal and middle meningeal artery is embolized. After 3–5 days, resection is performed. iii) Autologous blood transfusion equipment is used during surgery. iv) Drilling is performed on each side of the sagittal sinus. The craniotomy is performed using a skull cutter with an automatic protection function, avoiding the use of the guide plate and wire saw as much as possible. v) The dura and sagittal sinus are carefully stripped prior to opening the bone flap. Sagittal sinus ruptures and damage to the adjacent brain tissue must be avoided. vi) The blood pressure is controlled during the surgery (9), with the systolic blood pressure maintained at <90 mmHg and between 70 and 80 mmHg when required.

Post-operative complications, including paralysis, coma, cerebral edema and hemorrhagic infarction, are often associated with venous system injuries (10,11). Microsurgery is an important method for the complete resection of parasagittal meningioma. During the surgical procedure, arteries providing the blood supply must be treated first, followed by the treatment of the draining veins (12–14). Resections must be conducted following the blockage of the sagittal sinus blood supply. The Rolandic and draining veins and the peritumoral brain tissue must be protected effectively, and the associated sagittal sinuses must be treated correctly. These parameters are the key to a successful surgery. A segment of draining veins is located under the dura mater and must be protected when the dura mater is cut. Tumor tissues infiltrated by the Rolandic veins must be removed by block excision. In the present study, following the reduction of the tumor mass and intravenous tension, the Rolandic vein was stripped. As with three cases from teh present study, a small amount of tumor tissue may remain as it is extremely difficult to separate the tissue from the Rolandic vein.

All patients in the present study had large tumors. Block excision with brain tumor forceps was conducted to remove the tumor. Following a reduction in the tumor size and intravenous tension, the tumor capsule was completely removed. During the separation process, the peritumoral normal brain tissue and the cerebral pia mater must be effectively protected, and the aspiration method, which may damage the central gyri, must not be performed (12–14). In cases where the Rolandic vein is damaged, venous anastomosis or autogenous vein grafting must be performed (15). In the present study, the Rolandic and draining veins in one patient were identified to infiltrate the tumor tissue and run under the dura mater for a distance of 2 cm (Figs. 1–2). Block excision was successfully conducted in this case (Fig. 3).

An appropriate treatment of the involved sagittal sinuses is extremely important for preventing the post-operative recurrence of tumors. A complete resection of the tumor with a reconstruction of the sagittal sinus remains a controversial process; lateral resection of the sagittal sinus wall followed by repair with the dura mater, or complete resection of the sagittal sinus followed by anastomoses with autologous vein or artificial vessel, have been proposed as suitable methods for this procedure. However, these measures may lead to higher disability and mortality rates (16).

In the present study, the sagittal sinus was treated in accordance with the following principles of treatment: i) In cases where the tumor has only invaded the outer wall of the sagittal sinus, electrocautery is performed on the attaching wall following tumor removal, accompanied by saline washes. ii) If the tumor has invaded the whole sinusoidal wall or entered the sinus cavity, the tumor tissue in the sinus cavity and the involved sinus wall are removed, under conditions of adequate blood source and controlled blood pressure. The direct suture is conducted on the small sinus wall gap. If the sinus wall gap is bigger, the reconstruction of the sinus wall is performed with the fascia or artificial dura mater, and gelatin sponges and biological glue are used to reinforce the sinus wall. iii) If the sinus cavity is completely blocked with a fine peripheral venous return compensation, the blocked section of the sagittal sinus is removed together with the tumor (17,18). During surgery, the peritumoral large draining vein is protected and the end of the vein is sutured without electrocautery. These measures may prevent thrombosis formation, which affects the return compensation of the anastomotic vein. iv) Residual tumor tissue is treated with γ-Knife or X-Knife radiotherapy to control tumor growth for an extended period of time (19,20). In the current study, 6 patients with residual tumors were treated with this method. No post-operative recurrence was found in 4 of these patients during the follow-up examinations.

The results of the surgical procedure performed in the present study include pre-operative imaging evaluations, skilled microsurgical techniques, effective protection of the Rolandic veins and treatment of the sagittal sinus, as well as the avoidance of damage to the cerebral cortex. Together, this protocol significantly increases the total tumor removal rate and decreases post-operative recurrence during the microsurgical treatment of parasagittal meningioma in the central gyrus region.
